# The Role of Developmental Assets in Gender Differences in Anxiety in Spanish Youth

**DOI:** 10.3389/fpsyt.2022.810326

**Published:** 2022-04-25

**Authors:** Diego Gomez-Baya, Jose A. Salinas-Perez, Alvaro Sanchez-Lopez, Susana Paino-Quesada, Ramon Mendoza-Berjano

**Affiliations:** ^1^Department of Social, Developmental and Educational Psychology, Universidad de Huelva, Huelva, Spain; ^2^Department of Quantitative Methods, Universidad Loyola Andalucía, Seville, Spain; ^3^Department of Personality, Evaluation and Psychological Treatment, Universidad Complutense de Madrid, Madrid, Spain; ^4^Department of Clinical and Experimental Psychology, Universidad de Huelva, Huelva, Spain

**Keywords:** developmental assets, identity, values, anxiety, gender, emerging adulthood, youth

## Abstract

Anxiety disorders are the most prevalent psychological disorders during emerging adulthood. Some consistent gender differences have been reported in anxiety with women suffering more anxiety than men, which has detrimental consequences in most life spheres in the youth and later life stages. The understanding of the development of anxiety in emerging adulthood requires a developmental perspective. The Developmental Assets Theory was postulated to describe the individual and the contextual resources which may foster positive youth development and mental health. The present study aims to analyze to what extent the gender differences in anxiety may be partly explained by gender differences in developmental assets. For this purpose, a cross-sectional study was conducted in which a sample of 1,044 youths (75.5% women; age range = 18–28; M age = 20.47, SD = 3.08) enrolled in 11 universities from different regions in Spain filled in self-report measures of developmental assets and anxiety symptoms. The participants completed an online survey with the scales, Developmental Assets Profile developed by the Search Institute (1) and Generalized Anxiety Disorder-7 (GAD-7) (2). The results showed more anxiety in the female subsample (at both the symptoms and clinical levels). Some gender differences in developmental assets were also observed. A partial mediation model, based on regression analyses, indicated that gender differences in anxiety were partly explained by gender differences in developmental assets. Thus, higher anxiety in the women was partly due to lower scores in positive identity and higher scores in positive values. These results suggested the need to design programs to prevent anxiety with specific measures for women youth to nurture positive identity and promote strengths and coping skills that allow them to get the benefits of well-being derived from positive values, thus, preventing worry and stress overload, which may lead to anxiety.

## Introduction

Anxiety disorders are the most prevalent psychological disorders across development (3–5) and the most prevalent during emerging adulthood (6–8). In the United States, the lifetime prevalence is 32.4% in adolescence (13–17 years old) and 33.7% in adulthood (18–64 years old), with a 12-month prevalence of 25.2 and 21.3%, respectively (9). Polanczyk et al. (10) conducted a meta-analysis with 41 studies from 27 countries and found that the worldwide prevalence of any anxiety disorder was 6.5% (CI 95% 4.7–9.1) in children and adolescents. The recent results from the WHO World Mental Health International College Student in 19 universities across 8 countries (Australia, Belgium, Germany, Mexico, Northern Ireland, South Africa, Spain, United States) revealed that generalized anxiety disorder presented a lifetime prevalence of 18.6%, a 12-month prevalence of 16.7%, and a mean age of onset in 14.6-year-olds (11). Furthermore, the subthreshold expressions of anxiety disorders are also very common, and they increase the risk for threshold conditions (specifically, an occurrence over three times higher than among the respondents without subthreshold anxiety) (12). The onset of anxiety has been established in transition to adulthood, although different patterns across development have been observed in specific forms of anxiety: phobias and social anxiety seemed to begin in adolescence (between 15 and 17 years) while the onset of panic disorder and generalized anxiety have their onset during the youth, between 23 and 30 years (9). Beesdo-Baum and Knappe (13) found in a German sample that the age of onset of most anxiety disorders is during adolescence and youth, after performing a 5-year longitudinal study.

Furthermore, research indicates that anxiety disorders are more pervasive and persistent in adolescents and young people, although there is substantial continuity in the subsequent life stages in the presence of other anxiety disorders or other psychological disorders (14–16) that occur later. The presence of anxiety itself is also a risk factor for other psychopathologies, such as depression and substance abuse (17, 18). Anxiety has detrimental developmental consequences in most life spheres. Kessler et al. (19) showed that given the early age of onset, anxiety has negative effects on critical developmental transitions with important damages in education (associated with school dropout and failure to pass to higher education), marital timing and stability (i.e., lower probability of marriage or marriage before 18), marital quality (with more marital dissatisfaction, and an elevated risk for violence and victimization), childbearing (in concrete, more risk for teen childbearing and presence of negative parenting behaviors and maladaptive interactions), and occupation (e.g., higher unemployment, job loss, and more difficulties in job seeking and retention, which may be related to lower financial success). Furthermore, suffering from anxiety also increased the risk for physical illnesses, such as arthritis, asthma, hypertension, and heart disease (20).

## Gender Differences in Anxiety in the Emerging Adulthood

Some consistent gender differences have been reported in anxiety (21). While in childhood, no consistent gender differences were detected in anxiety (22), these differences seemed to emerge in the youth period. In the United States, Ohannessian et al. (23) conducted a prospective study over 2 years and found that in adolescent girls, generalized anxiety and social anxiety increased from middle to late adolescence, while the boys showed stable patterns. In Canada, Duchesne and Ratelle (24) performed a longitudinal study with six waves from 11 to 16-year-olds and concluded that the existence of gender disparities in anxiety with a subgroup of girls with moderate symptoms progressively increased over time. Faravelli et al. (25) and McLean et al. (26) indicated that the lifetime prevalence of anxiety disorders was approximately doubled in females.

Emerging adulthood is a transitional stage from adolescence to adulthood during the ages of 18–29 years, representing a prolonged period of exploration and change, new challenges, and opportunities (27). It has been indicated as the most unstable period with the most frequent changes in love and work as well as a highly self-focused life with fewer daily social roles and obligations to others (28). As such, emerging adulthood is a critical period for anxiety symptomatology. Anxiety disorders affect ~ 22.3% of individuals during this life stage (6). Previous epidemiological studies with emerging adults had concluded that the high prevalence rates of specific phobias, social anxiety, and adult separation anxiety may be due to an early onset in childhood or adolescence, while panic disorder, generalized anxiety, and agoraphobia began in this period (29). In emerging adulthood, some specific detrimental consequences are described in home management, college-related work, close relationships, and social life (30) as well as increasing the risk for anxiety and depression in later adulthood (31). In Spain, data from the Health National Survey in 2017 indicated that in the sample aged 15–24, 1.1% of men and 3% of women suffered from anxiety, while in the sample aged 25–34, this percentage increased to 3.6% in men and 5.9% in women (32). During the COVID-19 pandemic, an increase in anxiety has been observed in the youth population (33–35). Racine et al. (36) have concluded, in a recent meta-analysis of 29 studies including 80,879 youths globally, that the prevalence of anxiety symptoms during the pandemic has doubled, with 1 in 5 youths experiencing clinically elevated anxiety symptoms. Data from the European Health Survey in Spain in 2020 have shown that in the population aged 15–24, 2.07% of men and 4.15% of women reported anxiety, while these percentages in the population aged 25–64 were 4.31 in men and 8.50 in women (37).

## Developmental Assets Framework to Understand Gender Differences in Anxiety

The understanding of the development of anxiety in emerging adulthood requires a developmental psychopathology perspective. The transition to adulthood is a sensitive period in which there are important developmental tasks to accomplish (38). To explain the emergence of anxiety in youth, some risk factors have been described by the literature (38, 39), such as childhood adversity (e.g., parental mental illness, family violence, physical abuse, and economic adversity), insecure attachment, personality (e.g., shyness and neuroticism), experimentation with drugs, and social media use. Furthermore, more research is needed concerning the protective factors for the well-being of the youth. Derived from the relational developmental systems theory, the Developmental Assets Theory was postulated to describe the individual and contextual resources which may foster positive youth development, and are subsequently related to thriving indicators, such as positive mental health (1, 40, 41).

Developmental assets can be generalized across different social locations and represent factors on both the ecological and individual levels, which can be promoted by contexts and reached by young people through their interactions (42, 43). Two types of resources are presented in this model, i.e., internal and external assets (43, 44). The external assets include support (family support, positive family communication, other adult relationships, caring neighborhood, caring school climate, and parent involvement in schooling), empowerment (community values the youth, youth as a resource, service to others, and safety), boundaries and expectations (family, school, and neighborhood boundaries, adult role models, positive peer influence, and high expectations), and constructive use of time (creative activities, youth programs, religious community, and time at home). The internal assets entail a commitment to learning (achievement motivation, school engagement, homework, bonding to school, and reading for pleasure), positive values (caring, equality, and social justice, honesty, responsibility, and restraint), social competence (planning and decision making, interpersonal competence, cultural competence, resistance skills, and peaceful conflict resolution), and positive identity (personal power, self-esteem, sense of purpose, and a positive view of personal future).

These developmental assets are cumulative for promoting well-being and for reducing risk behaviors (43) so that a positive development occurs when adaptive individual-context interactions are built. In a study in Albania, Kosovo, Macedonia, and Serbia, the internal assets were positively associated with academic achievement and negatively with risky behaviors (45). In Norway, internal assets were negatively related to sadness and suicide attempts (46). In a study of youth from Ghana, Kenya, and South Africa, the internal assets were positively related to academic achievement (47). In Colombia and Peru, developmental assets were found to be a protective factor for substance-use behaviors and mental health (48). Bleck and DeBate (49) found that developmental assets predicted substance use and physical activity in a national longitudinal study from adolescence to adulthood in the United States. Smokowski et al. (50) found prospective evidence for the protective role of developmental assets for internalizing symptoms and self-esteem after a 5-year longitudinal study with a sample of disadvantaged adolescents in North Carolina (United States). In Portugal, developmental assets were positive predictors of life satisfaction in adolescents, explaining more than half of the variance (51).

Concerning gender differences in developmental assets, recently, Gomez-Baya et al. (52) found that female students showed higher scores in support, empowerment, boundaries and expectations, commitment to learning, positive values, and social competence, while the male students showed higher positive identity. These authors concluded that gender differences in developmental assets partly explained gender differences in positive youth development in a sample of adolescents and emerging adults in Andalusia (Spain). In Italy, Norway, and Turkey, Wiium et al. (53) found that Norwegian girls reported the highest number of developmental assets compared to the Norwegian boys and girls from the other participating countries. Also, in Norway, Issa et al. (54) concluded that the girls scored higher than the boys on the commitment to learning, positive values, social competence, support, and boundaries and expectancies, while the boys showed more positive identity.

## Study Justification and Aims

Since emerging adulthood is a crucial life stage in development, more research is needed to examine both the anxiety symptoms and generalized anxiety disorder, and their gender differences. Moreover, most evidence to date comes from studies from North American or North European countries, and research from other cultures and countries, such as Spain, is recommended to generalize the findings concerning gender differences in anxiety and its associated factors. Also, it is important to provide new results on youth anxiety during the COVID-19 pandemic.

To provide some guidelines for program design, the study of protective factors in emerging adulthood is also highly recommended to enhance competence development. Developmental assets present an interesting and comprehensive framework to explain the psychological adjustment in this transitional period to adulthood. In this vein, more research is needed to examine the associations between developmental assets and anxiety. Importantly, the integration between gender differences in both assets and anxiety could be a promising research line. Thus, this study aimed to examine the relationships between gender differences in both developmental assets and anxiety.

Specifically, three aims may be differentiated: (a) to analyze gender differences in anxiety and developmental assets; (b) to study the relationships between developmental assets and anxiety, controlling for demographics, and (c) to analyze to what extent the gender differences in anxiety may be partly explained by gender differences in developmental assets. Some hypotheses may be derived from the literature previously commented on. First, women are expected to report more anxiety than men, and some gender differences are expected, especially in the internal assets. Second, developmental assets are expected to be negatively related to anxiety (at both the clinical level and symptomatology scores). Third, gender differences in developmental assets are expected to partly mediate the gender differences in anxiety.

## Methods

### Participants

A sample of 1,044 youths (75.5% women; age range = 18–28; M age = 20.47, SD = 3.08) participated in the study. These participants were enrolled from a total of 11 universities from different regions in Spain: the University of Huelva, Loyola University (Campuses of Seville and Cordoba), Complutense University of Madrid, University of Granada, University of Salamanca, University of La Laguna, University of Zaragoza, University of Santiago, Polytechnic University of Valencia, University of Valencia, and the University of Oviedo), selected by convenience, controlling for geographical distribution around the country, i.e., including universities from the North, South, West, East, Center, and island territory. The degrees and academic years were randomly selected in each university. Concerning the academic year distribution, 27.1% were in the 1st year, 28.5 in the 2nd year, 22.9% in the 3rd, and 21.5% in the 4–6th academic years. The degrees were distributed in four areas of the study: Arts and Humanities (13.5%), Sciences and Engineering (20.2%), Health Sciences (24.9%), and Social Sciences and Law (41.4%). Up to 95.2% reported Spanish nationality and 0.6% had double nationality. Most of the samples (53.8%) lived with their parents, while 23.2% cohabited with other students. Concerning the habitat, 33.9% lived in a city with a population of 300,000, 32.2% lived in a city with 50,001–300,000 inhabitants, while the rest of the samples lived in towns or rural areas. Most of the samples (63.5%) indicated that they did not look for a job and 19.5% worked occasionally.

### Data Collection Procedure

The sample completed an online survey composed of different measures of mental health and lifestyles in young people. They needed around 30 min to fill in the self-report measure on average. Data collection was conducted in the winter and spring of 2021. This research was approved by the University of Huelva's ethics board on 11 January 2019. The 11 universities contacted agreed to join in the research. All participants provided written informed consent. The participants did not receive any reward for being involved in the study.

After selecting the degrees and academic years randomly from each university, the research team contacted the professors of each university. The professors were invited to share the online survey with their students through e-mails or online teaching platforms. A total of 527 contacts were made with professors, who were invited to share the survey with their group of students. About 26.4% of the professors responded to our mail positively on the first contact (*n* = 139), while in a second mail, 18% agreed to participate (*n* = 70). Thus, a total of 209 professors (39.7% of the contacted professors) agreed to share the survey with their respective groups of students. If each group has an estimated size of ~50 students, the contacted population was about 10,450 undergraduates (thus, the response rate was around 10%). Regarding gender distribution of the professors implicated, 55.1% were women. In the overall sample of the students who participated in the study, 88.1% completed the survey without any omission or <10 omissions. The survey was implemented in the Qualtrics application and data were included in SPSS 21.0 for analysis.

### Instrument

Some demographic information, anxiety, and developmental assets were assessed with these two scales.

#### Developmental Assets

The Developmental Assets Profile developed by the Search Institute was used (1). This instrument was adopted for Spanish adolescents and youth by Gomez-Baya et al. (52) with good psychometric properties. It was composed of 58 items distributed in eight subscales which represented two categories, i.e., internal assets and external assets. The external assets' dimension had four subscales: support (7 items; e.g., “I have a family that gives me love and support”), empowerment (6 items; e.g., “I am given useful roles and responsibilities”), boundaries and expectations (9 items; e.g., “I have parents who urge me to do well in school/university”), and constructive use of time (4 items; e.g., “I am involved in creative things such as music, theater, or other arts”). The internal assets' dimension included: commitment to learning (7 items; e.g., “I am trying to learn new things”), positive values (11 items; e.g., “I am developing respect for other people”), social competence (8 items; e.g., “I am sensitive to the needs and feeling of others”), and positive identity (6 items; e.g., “I feel good about myself”). The responses to the statements presented a four-point Likert scale from *not at all or rarely* (1) to *extremely or almost always* (4). The mean scores were calculated for each of the eight subscales. Good factorial validity in the Spanish sample was concluded by Gomez-Baya et al. (52) in adolescents and emerging adults. Concerning reliability, in this study, acceptable internal consistency was observed in support (α = 0.82), empowerment (α = 0.70), boundaries and expectations (α = 0.73), commitment to learning (α = 0.75), positive values (α = 0.76), and positive identity (α = 0.83). Low reliability was detected for constructive use of time (α = 0.34) and social competence (α = 0.67).

#### Anxiety Symptoms

The Generalized Anxiety Disorder-7 (GAD-7) (2) was used. This scale presented good psychometric properties in adolescents and youth in Spain (55). This questionnaire was introduced with “How often have you been bothered by the following over the past 2 weeks?” and described seven items, which assessed different anxiety symptoms. The intensity of each symptom was evaluated following a 4-point-scale from 0 (not at all) to 3 (nearly every day). The overall score was calculated by adding scores ranging from 0 to 21. The literature to date has established cutoff points at 5, 10, and 15 for mild, moderate, and severe anxiety, respectively. Generalized anxiety disorder was calculated based on a cutoff point of 10. In this study, it has excellent reliability (α = 0.90).

### Data Analysis' Design

First, descriptive statistics (mean and SD) were presented for developmental assets and anxiety symptoms, and frequency distribution was examined for generalized anxiety disorder. The gender differences in developmental assets and anxiety symptoms were analyzed with the Student's *t*-tests, while the difference in generalized anxiety disorder was calculated with χ^2^. Second, two regression analyses were performed to respectively explain the anxiety symptoms and generalized anxiety disorder, controlling for demographics (gender, age, nationality, area of degree, and university) and including developmental assets as independent variables. A hierarchical regression analysis was carried out for anxiety symptoms, with two steps: the first one with demographics, and adding developmental assets in the second one. For generalized anxiety disorder (categorized as generalized anxiety disorder or none), a logistic regression analysis was performed with the same steps, including 95% CIs for the exponentiation of the B coefficient. These analyses were carried out with SPSS 21.0.

Third, a partial mediation model was tested. A regression-based macro for SPSS called Process v3.0, developed by Hayes (56), was used to perform these analyses, following the recommendations described by Preacher and Kelley (57). The total effect model represents the effect of gender on anxiety symptoms, while the mediational model examined the mediation of developmental assets in the relationship between gender and anxiety. Multiple partial mediations were implemented with 5,000 bootstrap samples estimated for bias-corrected bootstrap 95% CIs for specific indirect effects. Standardized variables were created to perform these analyses, and *F* statistics, *R*^2^ values, effect coefficients, CIs, and measurement errors were reported. The statistical significance was indicated at *p* < 0.05.

## Results

### Descriptive Statistics and Gender Differences

Table 1 presents the descriptive statistics and frequency distribution of the study variables in the overall sample and by gender. Concerning developmental assets, greater mean scores were observed in commitment to learning, positive values, empowerment, and social competence, while the lowest score was found in the creative use of time. The moderate mean score was detected for anxiety symptoms, while 42.1% of the sample presented a clinical score, following the cutoff point at 10. Some gender differences were found in these variables. First, women reported more anxiety symptoms than men, with a difference of two points. This tendency was also detected in the generalized anxiety disorder, with a difference of around 15% for the women subsample (women: 45.8%; men: 30.4%). Second, some differences were shown in developmental assets. Specifically, women reported more positive values, social competence, commitment to learning, expectations and boundaries, and support, while men reported more positive identity.

**Table 1:** Descriptive statistics and gender differences in anxiety and developmental assets.

	**Total (*n =* 1,044)**	**Women (*n =* 788)**	**Men (*n =* 256)**	**Gender differences**	
	**M(SD)**	**M(SD)**	**M(SD)**	***t***	**Cohen's d**
Anxiety	8.61 (5.42)	9.12 (5.51)	7.03 (4.82)	5.24^***^	0.40
Support	2.92 (0.61)	2.95 (0.61)	2.85 (0.61)	2.33^*^	0.16
Empowerment	3.26 (0.49)	3.27 (0.50)	3.25 (0.61)	0.37	0.04
Expectations and boundaries	2.84 (0.49)	2.86 (0.49)	2.77 (0.48)	2.44^*^	0.19
Creative use of time	2.25 (0.62)	2.23 (0.60)	2.28 (0.68)	−1.12	0.08
Commitment to learning	3.35 (0.48)	3.39 (0.45)	3.25 (0.54)	4.16^***^	0.28
Positive values	3.27 (0.38)	3.32 (0.36)	3.14 (0.39)	6.65^***^	0.48
Social competences	3.24 (0.41)	3.28 (0.40)	3.12 (0.42)	5.45^***^	0.39
Positive identity	2.75 (0.63)	2.72 (0.63)	2.84 (0.63)	−2.57^*^	0.19
	%	%	%	X	Phi
Clinical generalized anxiety disorder	42.1	45.8	30.4	17.57^***^	0.13

* *p <0.05*;

** * p <0.01*;

*** * p <0.001*.

### Regression Analyses

Table 2 describes the results of regression analyses to explain anxiety (both symptoms and clinical) based on developmental assets and controlling for demographics. In the first step, the results indicated only gender-explained anxiety symptoms and clinical severity. No effect was detected by age, nationality, area of degree, or university. This step reached a low-explained variance. In the second step, developmental assets were introduced and the explained variance increased to more than 20%. The anxiety symptoms results showed that gender, empowerment, commitment to learning, positive values, and positive identity had significant effects. The negative effects were detected by positive identity and empowerment, and the positive ones were found by positive values and commitment to learning. Concerning generalized anxiety disorder, as well as gender, positive identity and empowerment had negative effects and positive value presented a positive effect.

**Table 2:** Hierarchical regression analyses to explain anxiety and generalized anxiety disorder based on developmental assets.

	**Anxious symptoms**				**Generalized anxiety disorder**						
	***F***	***R* ^2^**	***t***	**β**	***X* ^2^**	***R* ^2^**	**Wald**	***B***	**Exp(B)**	**LLCI**	**ULCI**
1st step	7.01^***^	0.04			22.99^***^	0.03					
Gender			−5.41	−0.17^***^			18.10	−0.69^***^	0.50	0.37	0.69
Age			−1.27	−0.04			1.15	−0.02	0.98	0.94	1.02
Nationality			−1.29	−0.04			0.33	−0.15	0.86	0.51	1.45
Area of degree			−1.19	−0.04			0.82	−0.06	0.94	0.83	1.06
University			1.54	0.05			2.26	0.03	1.03	0.99	1.08
2nd step	26.54^***^	0.27			199.93^***^	0.25					
Gender			−2.91	−0.09^**^			5.35	−0.43^*^	0.65	0.45	0.94
Age			−1.45	−0.04			0.76	−0.02	0.98	0.93	1.03
Nationality			−0.21	−0.01			0.17	0.12	1.13	0.63	2.05
Area of degree			0.44	0.01			0.06	0.02	1.02	0.89	1.17
University			0.23	0.01			0.25	0.01	1.01	0.97	1.06
Support			0.24	0.01			0.15	0.04	1.05	0.84	1.30
Empowerment			−5.34	−0.23^***^			19.94	−0.51^***^	0.60	0.48	0.75
Expectations and boundaries			−0.26	−0.01			0.12	0.04	1.04	0.83	1.30
Creative use of time			0.47	0.02			1.00	0.09	1.09	0.92	1.29
Commitment to learning			2.42	0.08^*^			3.36	0.16	1.17	0.99	1.39
Positive values			4.45	0.17^***^			12.54	0.36^***^	1.44	1.18	1.76
Social competences			0.01	0.01			0.46	−0.07	0.93	0.77	1.14
Positive identity			−11.53	−0.42^***^			74.12	−0.87^***^	0.42	0.35	0.51

* *p <0.05*;

** * p <0.01*;

*** * p <0.001. LLCI, Low Level Confidence Interval; ULCI, Upper Level Confidence Interval*.

### Partial Mediation Analysis

**Figure 1:**
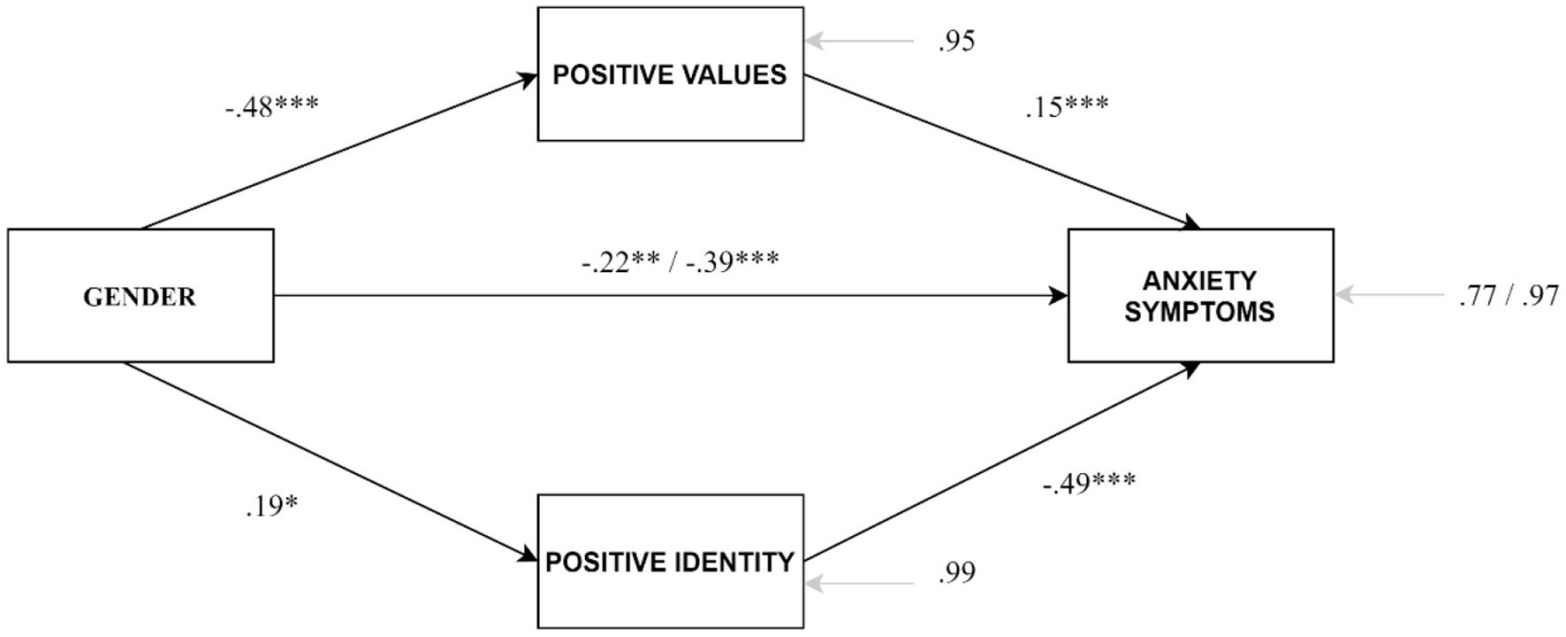
Partial mediation model to examine the partial mediation of positive values and positive identity in the effect of gender on anxiety symptoms. *p < 0.05; **p < 0.01; ***p < 0.001.

Table 3 shows the results of the partial mediation model to explain gender differences in anxiety symptoms based on gender differences in developmental assets. Despite empowerment having a significant effect in explaining anxiety, it was not introduced in the model because no gender differences in that asset were observed. Thus, commitment to learning, positive values, and positive identity was included in the regression model to examine the partial mediation between gender and anxiety symptoms. Significant gender effects were observed on the three mediators. Concerning the effects on the anxiety symptoms, the results indicated that positive values and positive identity had a significant effect. The gender maintained a significant effect, while the effect of commitment to learning was not significant. Compared to the total effect model, the model including the mediators reached an increase in the explained variance of 20%, and the magnitude of the effect by gender was half reduced. The model indicated that gender differences in the anxiety symptoms were partially due to gender differences in positive values and positive identity. In concrete, lower scores in positive identity and higher scores in positive values partially explained the greater presence of anxiety symptoms in women youth. Figure 1 represents the final model after deleting the non-significant effect of the commitment to learning. The gender's effect on the anxiety symptoms was partially mediated by positive values (indirect effect = −0.07, bootSE = 0.02, BootLLCI = −0.11, BootULCI = −0.04) and positive identity (indirect effect = −0.09, bootSE = 0.04, BootLLCI = −0.17, BootULCI = −0.03), *F*_(3, 975)_ = 93.08, *p* < 0.001, *R*^2^ = 0.23.

**Table 3:** Multiple partial mediation model with anxiety symptoms.

	***F***	***R* ^2^**	**MSE**	***t***	**β**	**LLCI**	**ULCI**
**Direct effect model**
DV: Commitment to learning	15.71^***^	0.02	0.988				
Gender				−3.96	−0.32^***^	−0.49	−0.16
DV: Positive Values	41.27^***^	0.04	0.950				
Gender				−6.42	−0.48^***^	−0.63	−0.33
DV: Positive identity	6.72^*^	0.01	0.994				
Gender				2.59	0.19^*^	0.05	0.34
DV: Anxiety symptoms	69.62^***^	0.23	0.772				
VI: Gender				−3.23	−0.20^**^	−0.33	−0.08
IV: Commitment to learning				1.56	0.05	−0.01	0.12
IV: Positive values				4.14	0.14^***^	0.07	0.20
IV: Positive identity				−14.66	−0.51^***^	−0.58	−0.44
**Total effect model**	31.58^***^	0.03	0.974				
Gender				−5.62	−0.39^***^	−0.52	−0.25

* *p <0.05*;

** *p <0.01*;

*** *p <0.001. LLCI, Low Level Confidence Interval; ULCI, Upper Level Confidence Interval; VD, Dependent variable; IV, Independent variable*.

## Discussion

This research with a sample of Spanish emerging adults presented three aims. The first aim was to examine the differences by gender in anxiety and developmental assets. The results have indicated that the female youth reported more anxiety symptoms on average, and a great percentage of female youth reached generalized anxiety disorder compared to the male youth. These results are consistent with our hypothesis and with the results of 25 and McLean et al. (26). The percentage of the participants who presented a mean score over the cutoff point for generalized anxiety disorder was higher than the results observed in previous research with youth, especially in the sample of women. Furthermore, as expected and in line with the studies by Gomez-Baya et al. (52) and Issa et al. (54), some gender differences were also identified in the developmental assets. In this study, the results showed that women had greater scores in two external assets (i.e., support and expectations/boundaries) and three internal assets (i.e., commitment to learning, positive values, and social competence). However, higher scores were detected in the internal asset of positive identity in the subsample of men. These differences could be explained based on gender socialization in adolescence and youth, so that the gender role stereotypes may guide the development of social expectations, attitudes, and behaviors (58). Josephs et al. (59) concluded that the feeling of self-worth in men is linked more to autonomy and personal achievements, while the self-worth in women depends on connection and sensitivity to others. In this vein, the higher satisfaction of women with interdependent areas of their lives may encourage more altruistic and caring behaviors (60). Previous research has already pointed out that girls develop more academic motivation than boys (61) and more school engagement (62), while they present lower self-esteem (63). Consequently, it seems that gendered socialization makes more accessible the resources regarding social interdependency to females, while achievement and positive identity are more encouraged in men.

The second aim was to examine the relationships between developmental assets and anxiety. In line with the Developmental Assets Theory (40), more presence of assets was expected to be associated with thriving, translated into better health and mental health results, as shown by Manrique-Millones et al. (48), Wiium et al. (46), and Smokowski et al. (50). As expected, the results showed that empowerment and positive identity were the assets (external and internal, respectively), which had a negative significant effect on anxiety, at the clinical and symptomatology levels. However, positive values were found to be positively related to anxiety. The measure of positive values included indicators such as: “I think it is important to help other people,” “I am helping to make my university, neighborhood or city a better place,” or “I am serving others in my community” (1). This paradoxical result may be explained because the youth with more positive values feel encouraged to pay attention and take care of others as well as work for improving their communities. If they have not yet developed the correct strengths and coping strategies to deal with stress and problems, they may experience increased worry and anxiety while trying to fight against the relational and contextual problems. Recently, Kozina et al. (64) have observed, in a sample of adolescents and youth from Slovenia and Spain, that the dimension of positive youth development of caring, which is characterized as a feeling of sympathy and empathy for others, has a positive association with anxiety. These authors argued that people who are characterized as more caring would have difficulties with emotional contagion, which may subsequently impair their attempt to help others. Thus, more caring would not be necessarily related to more well-being if the person is not prepared enough to deal with that situation, which may lead to increased worry and helplessness. Shu et al. (65) underlined the impact of social environment on experiencing vicarious anxiety in these people with high empathy.

Finally, the third aim was to examine if gender differences in developmental assets explained gender differences in anxiety. The results indicated that higher anxiety in women is partly due to lower scores in positive identity and higher scores in positive values. These results are the main contribution of the present study to explain gender differences in anxiety based on gender differences in developmental assets. The association between positive identity and anxiety is consistent with studies with close variables such as self-efficacy (66) and self-esteem. A meta-analysis of the longitudinal studies by Sowislo and Orth (67) concluded that low self-esteem predicted anxiety. Thus, low self-esteem may be a risk factor for anxiety, but high self-esteem may serve as an anxiety-buffering function too (68). A stable and positive self-image may be associated with well-being, achievement, and laudable interpersonal behavior (69). Furthermore, the relationship between positive values (related to caring, equality and social justice, integrity, honesty, responsibility, or restraint) and anxiety maybe explained due to stress and worry overload, in a developmental period in which some other challenges are faced and some competence and coping skills need to be developed on the way to adulthood. MacDonald and Price (70) have observed that difficulties in emotion regulation mediated the relationship between affective empathy and internalizing symptoms. Zalta and Chambless (71) concluded that instrumentality and mastery mediate the relationship between gender and anxiety after controlling the effects of daily hassles and social desirability. In line with the research about caregiver stress and mental health, women suffered more detrimental consequences for their mental health (72). Women had higher levels of burden and lower levels of well-being and physical health (73). Moreover, some differences in prosocial behaviors have been pointed out between men and women. Eagly (74) argued that women were more likely to engage in prosocial behaviors on a relational dimension, whereas men preferred an independent and strength-intensive dimension. Reis et al. (75) observed in the Portuguese youth that women reported more general worry than men, and it was associated with more anxiety symptoms. Regarding youth activism, expending large amounts of effort actively engaging in changing the structural conditions might have negative consequences on health (76). Regarding the amount of time that the youth spent helping their families (e.g., cooking, cleaning, and sibling care), some evidence indicated relationships with poorer health in the cases of burden overload (77).

### Study Limitations

Despite the results described in this research, some limitations should be acknowledged. Because self-report measures have been used, data may be biased by the subjective assessment or by social desirability. Moreover, two subscales of developmental assets did not present acceptable internal consistency (i.e., constructive use of time and social competence). The other developmental assets' subscales and the scale for anxiety symptoms reached notable reliability scores. A multi-method strategy could be recommended as a future research line to explore gender differences in assets and mental health. Also, other variables should be controlled in the examination of anxiety symptomatology. Because depressive symptoms co-occur frequently with anxious symptoms (78, 79), they could also be measured in future research. Furthermore, the results only draw associations between the study variables, and no directionality can be concluded. Longitudinal studies during emerging adulthood are recommended to examine how developmental assets predict anxiety and how gender differences emerge.

Other limitations come from the generalizability of the results within the Spanish context. The participating universities were selected by convenience and the access to the participants depending on the contact with the professors of each institution. Less than half of the professors contacted agreed to participate by sharing the survey with their students. Moreover, the response rate was very low among the students invited, so the results may be biased by this sampling procedure. Furthermore, most of the participants were women (75.5%), so it is necessary to improve the accessibility to male students. In Spain, there were around 1,293,576 undergraduates and 55.2% of them were women (80). This selection bias may explain the very high prevalence of generalized anxiety disorder (according to the cutoff point) observed in our sample, especially in the women subsample). Moreover, future research should examine those young people who are professionally training, are working or unemployed, to collect a more representative image of this population.

### Implications for Practice

The results may suggest some practical implications. The preventive measure to address anxiety in emerging adults needs to consider gender differences in the factors associated. To date, whereas widespread attention has been given to gender differences in anxiety and its causes (81, 82), less attention has been paid to differences in the prevention or treatment (83). The prevention of anxiety disorders in the youth may be more effective if the knowledge about gender specificity was used in the program design (83). Thus, programs should include measures to nurture positive identity (i.e., personal power, self-esteem, sense of purpose, and positive view of personal future) in the female youth. Also, programs should promote competence and coping skills to allow to reach the benefits for well-being derived from positive values, thus, preventing worry and stress overload, which may lead to anxiety. In general, empowerment, positive identity, and effective skills to put into practice the positive values could be innovative components for preventing anxiety in women during emerging adulthood based on asset nurturing. In this line, school-based prevention programs were found to be effective to prevent anxiety (84). Also, community programs have reached effectiveness (85). Among these interventions, the cognitive-behavioral therapy (CBT) ingredients were more common than the other interventions. These components may be complemented with a focus on positive youth development and asset development and with a gender perspective. Gallegos-Guajardo et al. (86) developed a 10-week program for selective prevention and resilience promotion in girls at risk. Based on the Friends program, a CBT-based resilience program, they found a decrease in anxiety symptoms and psychosocial difficulties as well as an increase in proactive coping skills. (87) performed a dance-based intervention to reduce internalizing problems in girls. They found that the intervention increased acceptance, self-trust, and emotional expression, which helped to improve their psychological adjustment.

Positive youth developmental programs were found to be effective to develop thriving in emerging adulthood (88). Following the review by (89), the promotion of competencies at the individual level and system level produces adaptive youth development outcomes (i.e., increase in prosocial behavior, academic achievement, and peer acceptance as well as reductions in negative behavior, violence, and peer rejection). Schaillée et al. (90) developed a program to promote positive youth development in girls through sports with positive results in personal and social skills. “Girls on the run” is another program with good results to promote girls' well-being through physical activity (91). Guerra and Bradshaw (92) linked the prevention of externalizing and internalizing problems with the promotion of well-being. In this line, Reis et al. (93) encouraged the implementation of the Health Promoting University, which focused on the promotion of healthy lifestyles and strength development to promote health and well-being in undergraduates. Newton et al. (94) noted “the importance of viewing the organization as a social system and of fostering and nurturing the “whole” by understanding and paying attention to the complex interactions and interconnections between component parts.” From a relational developmental systems perspective, mental health is the result of positive interactions between the individual and the context (40). Thus, the effective promotion of developmental assets in young males and females could be a thriving promotion strategy that could complement and boost traditional anxiety prevention programs. Regarding the positive values, the promotion of effective self-regulation and coping strategies may be recommended to gain well-being benefits from this asset, and prevent possible detrimental consequences (65).

## Conclusion

This research has examined gender differences in developmental assets and anxiety in emerging adults. The results highlighted that women reported more anxiety partly due to lower scores in positive identity and higher scores in positive values. The design of the anxiety prevention programs in female youth may foster a positive identity (e.g., self-esteem and personal power) as well as develop strengths and skills needed to take more well-being benefits from the positive values (e.g., caring and responsibility).

## Data Availability Statement

The raw data supporting the conclusions of this article will be made available by the authors, without undue reservation.

## Ethics Statement

The studies involving human participants were reviewed and approved by the bioethics committee of the University of Huelva. The patients/participants provided their written informed consent to participate in this study.

## Author Contributions

All authors listed have made a substantial, direct, and intellectual contribution to the work and approved it for publication.

## Funding

This research received funding from Research, Development and Innovation Projects of European Regional Development Fund in Andalusia 2014–2020. Grant number UHU-1259711, awarded to the first and last authors.

## Conflict of Interest

The authors declare that the research was conducted in the absence of any commercial or financial relationships that could be construed as a potential conflict of interest.

## Publisher's Note

All claims expressed in this article are solely those of the authors and do not necessarily represent those of their affiliated organizations, or those of the publisher, the editors and the reviewers. Any product that may be evaluated in this article, or claim that may be made by its manufacturer, is not guaranteed or endorsed by the publisher.
